# Cholesterol-Bearing Polysaccharide-Based Nanogels for Development of Novel Immunotherapy and Regenerative Medicine

**DOI:** 10.3390/gels10030206

**Published:** 2024-03-18

**Authors:** Tetsuya Adachi, Yoshiro Tahara, Kenta Yamamoto, Toshiro Yamamoto, Narisato Kanamura, Kazunari Akiyoshi, Osam Mazda

**Affiliations:** 1 Department of Dental Medicine, Graduate School of Medical Science, Kyoto Prefectural University of Medicine, Kamigyo-ku, Kyoto 602-8566, Japan; yamamoto@koto.kpu-m.ac.jp (T.Y.); kanamura@koto.kpu-m.ac.jp (N.K.); 2 Department of Immunology, Graduate School of Medical Science, Kyoto Prefectural University of Medicine, Kamigyo-ku, 465 Kajii-cho, Kyoto 602-8566, Japan; fiori30@koto.kpu-m.ac.jp (K.Y.);; 3 Department of Chemical Engineering and Materials Science, Doshisha University, 1-3 Tatara Miyakodani, Kyoto-fu, Kyotanabe-shi 610-0321, Japan; ytahara@mail.doshisha.ac.jp; 4 Department of Polymer Chemistry, Graduate School of Engineering, Kyoto University, Katsura, Nishikyo-ku, Kyoto 615-8510, Japan; akiyoshi.kazunari.2e@kyoto-u.ac.jp

**Keywords:** nanogel, drug delivery system, scaffold

## Abstract

Novel functional biomaterials are expected to bring about breakthroughs in developing immunotherapy and regenerative medicine through their application as drug delivery systems and scaffolds. Nanogels are defined as nanoparticles with a particle size of 100 nm or less and as having a gel structure. Nanogels have a three-dimensional network structure of cross-linked polymer chains, which have a high water content, a volume phase transition much faster than that of a macrogel, and a quick response to external stimuli. As it is possible to transmit substances according to the three-dimensional mesh size of the gel, a major feature is that relatively large substances, such as proteins and nucleic acids, can be taken into the gel. Furthermore, by organizing nanogels as a building block, they can be applied as a scaffold material for tissue regeneration. This review provides a brief overview of the current developments in nanogels in general, especially drug delivery, therapeutic applications, and tissue engineering. In particular, polysaccharide-based nanogels are interesting because they have excellent complexation properties and are highly biocompatible.

## 1. Introduction

**Figure 1:**
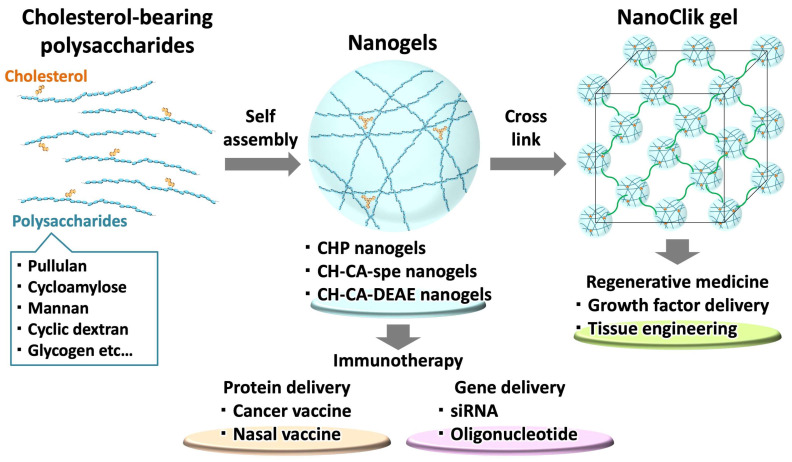
Schematic illustration of this review. Cholesterol-substituted polysaccharides form self-assembled nanogels that are cross-linked to build a nanogel-cross-linked (NanoClik) gel. Nanogels are used in immunotherapy, delivering proteins and genes. NanoClik gels are used in regenerative medicine, delivering growth factors or adhering cells for tissue engineering.

Novel functional biomaterials are expected to bring about breakthroughs in the development of immunotherapy and regenerative medicine through their application as drug delivery systems (DDSs) and scaffolds. We developed a nanogel using self-organizing cholesteryl pullulan (CHP). This is a water-soluble, non-ionic natural polysaccharide with many hydrophilic functional groups and is partially introduced with a hydrophobic functional group, a cholesterol group, in water (Figure 1).

In CHP nanogels, cholesterol groups associate with each other through hydrophobic interactions in an aqueous solution to form a network structure, which swells in water to form a spherical hydrogel structure [1]. The CHP nanogel has been developed and applied as a nanocarrier for protein DDSs in particular. In addition, various polysaccharides, such as cycloamylose, mannan, cyclic dextran, and glycogen [2], have been used as main chains and modified with cholesterol groups to form nanogels. Proteins, such as antigens for vaccines, cytokines, and hormones, can be encapsulated and released in vivo. To deliver nucleic acids, cholesterol-bearing polysaccharide-based nanogels were modified with cationic groups such as amine or spermine. These cationic nanogels were effective for gene delivery.

Furthermore, by organizing nanogels as a building block, they can be applied as a scaffold material for tissue regeneration [1]. In this paper, we introduce the application and development of highly functionalized nanogels for immunotherapy and regenerative medicine. In recent years, protein-based biopharmaceuticals, such as cytokines, monoclonal antibodies, and recombinant vaccines, have effectively treated various diseases. As biologics are unstable in vivo, it is difficult to maintain their effects for a long time. To solve these problems, there is a need to develop a protein stabilization technology and a delivery system that can be released over a long time in the affected area. Nanogels have been developed as protein nanocarriers. These polysaccharide nanogels contain and stabilize proteins in a self-organizing manner, inhibiting their aggregation and retaining their activity against various external stimuli. The nanogels are molecular chaperones that can release proteins [3].

Many types of nanoparticles have been used for drug delivery. These types have been classified by their components, such as lipid-based nanoparticles (e.g., liposomes, lipid nanoparticles, and emulsions), inorganic nanoparticles (e.g., quantum dots, silica, iron oxide, and gold nanoparticles), and polymeric nanoparticles (e.g., polymersomes, polymer micelles, and nanospheres) [4]. In preparing standard-type liposomes [5], lipids are self-assembled in water to form bilayers by hydrophobic interactions, and spherical vesicles are built from these lipid-based bilayers.

Similarly, in typical polymersomes [6], amphiphilic block polymers are self-assembled to form polymer-based bilayers, and spherical vesicles are made. In both liposomes and polymersomes, shells consisting of a single or multiple bilayers are formed around the inner water phase. In another case, amphiphilic block polymers are self-assembled in water to form polymer micelles [7], in which a condensed polymer core is made in the center, and hydrophilic polymer chains form a shell or corona of nanoparticles.

In most polymer nanospheres and inorganic nanoparticles, the starting materials are insoluble in water and form spherical nanoparticles in water. A nanogel is one subclass of polymeric nanoparticles, and its structure differs from polymersomes or polymeric micelles. In 2007, a nanogel was defined by the International Union of Pure and Applied Chemistry [8] as a nanometer-sized gel. In a previous study, the molecular weight of a single nanoparticle consisting of CHP was measured accurately by size exclusion chromatography with multiangle laser light scattering. It was confirmed that CHP polymers were assembled spontaneously through hydrophobic interactions in water and formed physically cross-linked hydrogel nanoparticles [9].

Additionally, the results of small-angle neutron scattering measurements were reported in 2016. It was shown that there are 19 cross-linking points in one CHP nanogel with an average radius of 8.1 nm, and the distance between the cross-linking points was 1.7 nm on average [10]. These results suggested that the inner water phase or core–shell structure was not formed in CHP-based nanogels, and different drug delivery approaches are possible compared with the other nanoparticles. This review will also introduce application examples of CHP nanogels used in DDS and scaffolds for regenerative medicine and comparisons with the various polymers (nanomicelles, lipid nanoparticles, and collagens).

## 2. Nanogels and Immunotherapy

### 2.1. Delivery of Antigens and Adjuvants

Nanogels can be incorporated into cells and have excellent properties as nanocarriers. Furthermore, polysaccharide nanogels can be used in cancer vaccine therapy by efficiently presenting cancer antigens to immune cells. By delivering an adjuvant (the TLR9 ligand CpG deoxynucleotides or the TLR3 ligand poly-IC) to immune cells (macrophages and dendritic cells) localized in tumor tissue using nanogels, the tumor microenvironment is immunologically activated to enhance the antitumor effect [11].

CHP nanogels improve the efficacy of cancer vaccines consisting of relatively large molecular weight proteins, efficiently activating both antigen-specific cytotoxic T cells and helper T cells, as demonstrated in clinical trials [12].

Kiyono’s group reported that nasal administration of a complex of Hc proteins and a cationic CHP nanogel (cCHP) with ethylenediamine groups significantly increased the production of IgG and IgA antibodies [3,13]. Using cCHP, it has become possible for antigens to remain in the nasal mucosa for a relatively long period and continuously penetrate through the nasal epithelial cell layer. cCHP has excellent properties as a carrier for nasal vaccines and is expected to be used as a new needle-free vaccine for various virus infection diseases, including coronavirus disease 2019 (COVID-19).

### 2.2. Gene Delivery

Nucleic acid drugs are attracting attention as a new cancer immunotherapy following antibody drugs. Due to the rapid spread of mRNA vaccines against COVID-19 [14], nucleic acid medicines are expected to be used as therapeutic agents for various diseases, including cancer.

The effectiveness and safety of mRNA vaccines against COVID-19 have been demonstrated, and research on mRNA-based cancer therapy and regenerative medicine is currently being carried out worldwide. As there is no risk of mRNA being inserted into the chromosomes, it can be used safely regarding the potential risk of tumorigenesis caused by aberration of genomic integrity. Since mRNA is extremely unstable in vivo, a DDS is required to stably retain and transport mRNA to target cells. In vivo, when mRNA or plasmid DNA (pDNA) was administered to skeletal muscle and protein expression was compared, the amount of protein expression by mRNA was lower than that by pDNA [15]. The reason for this is thought to be that mRNA is extremely unstable in vivo, and the use of pDNA has long been considered promising for gene therapy applications.

Katalin Karikó reported that replacing “uridine” with “pseudouridine” controlled the inflammatory response and dramatically increased the synthesis efficiency of the target protein [16,17,18]. Karikó and Drew Weissman won the 2023 Nobel Prize in Physiology or Medicine for developing the fundamental technology for mRNA vaccines [19,20].

Lipid nanoparticles (LNPs) have been widely used in COVID-19 vaccines [21]. The construction of LNPs for mRNA delivery is very important and was developed and published before 2019. Distearoylphosphatidylcholine (DSPC) and polyethylene glycol (PEG)-conjugated lipids were used in LNPs like traditional liposomes. A novel biodegradable amino lipid with a surface pKa < 7 was developed for efficient mRNA delivery [22]. These key materials were used in COVID-19 vaccines, but no conclusion has been reached regarding the optimal carrier for mRNA delivery.

Furthermore, compared with infectious disease vaccines, cancer vaccines are difficult to develop because cancer cells are difficult to distinguish from normal cells and have immunosuppressive effects. Using their own RNA engineering techniques, Tockary et al. developed a method to incorporate adjuvants directly into the mRNA strand that encodes the antigen without interfering with the ability to produce the antigen protein [23]. They designed short double-stranded RNA (dsRNA) targeting the innate immune receptor retinoic acid-inducible gene-I (*RIG-I*) [24] and interdigitated it with the mRNA strand through hybridization. They discovered a Comb-structured mRNA that efficiently stimulated RIG-I by changing the length and sequence of dsRNA. The resulting Comb-structured mRNA effectively activated dendritic cells, which are important in obtaining vaccine efficacy. In addition, by carrying Comb-structured mRNA not only in LNP but also in the polymeric nanomicelles that Kataoka developed [25], Tockary et al. succeeded in improving the efficacy of vaccines.

Polymer nanomicelles are nanoparticles formed by the self-association of a block copolymer composed of two segments: a hydrophilic polymer (such as PEG) and a hydrophobic polymer (such as a polyamino acid derivative). They have a two-layered structure in which the inner core is covered with an outer shell of a hydrophilic polymer.

The mRNA delivery system using polymeric micelles has also been applied to regenerative medicine. Aini et al. suppressed the progression of osteoarthritis by administering polymeric micelles containing mRNA for RUNX1. This transcription factor plays a key role in cartilage formation in the knee of an osteoarthritis model [26]. The effect of Runx2 was improved by administering mRNA for an osteoinductive transcription factor (Runx2), which has a bone regeneration effect. Further, mRNA was administered for VEGF, a cytokine that plays an important role in angiogenesis, into the bone defects [27]. There are still only a few examples of the application of mRNA medicine to regenerative medicine, and the only clinical trial currently being carried out in the world is the treatment of ischemic heart disease using *VEGF* mRNA, so future developments are currently attracting attention [28,29].

As well as mRNA delivery, the RNA interference (RNAi) method using small interfering RNA (siRNA) and small hairpin RNA (shRNA) can efficiently suppress specific genes, and its design is simple and versatile. However, there are still many unknowns about the administration method of siRNA to living tissue and the safety of the administered siRNA in vivo.

Developing a DDS carrier made of a biocompatible substance has been challenging when establishing a safe administration method for siRNA with few side effects. As cancer growth requires angiogenesis to supply nutrients to cancer cells, vascular endothelial growth factor A (VEGF-A) is produced in the tumor microenvironment, promoting vascular endothelial proliferation and angiogenesis [30,31].

New blood vessels in tumor tissue have a fragile structure. It is known that the extravasation of oxygen and chemotherapeutic agents carried through the bloodstream prevents sufficient therapeutic effects from being obtained [32]. Therefore, controlling angiogenesis in the tumor microenvironment will improve the efficacy of cancer therapy.

We developed a cholesterol-bearing cycloamylose modified with spermine (CH-CA-Spe) nanogel. We administered a complex of siRNA specific for VEGF-A (siVEGF) and the nanogel into the tumor tissue of a mouse renal cancer subcutaneous transplantation model. When the FITC-siRNA/nanogel complex was injected into the tumor of a mouse renal cancer subcutaneous transplantation model, it was confirmed that FITC-siRNA was maintained in the tumor tissue for a long period. It was also confirmed that administration of the siRNA/nanogel complex targeting VEGF-A resulted in a knockdown effect of VEGF gene expression in the tumor tissue. Continuous administration significantly inhibited the growth and angiogenesis of subcutaneously implanted tumors [33]. We also found that an intratumoral administration of the siVEGF/nanogel complex significantly suppressed the accumulation of myeloid-derived suppressor cells (MDSCs) [34,35] in the spleen compared with the control group.

These results suggest that VEGF-A expression is successfully knocked down in tumor cells by an intratumoral administration of the siVEGF/nanogel complex, suppressing tumor growth and restoring immunosuppression induced by the tumor [34,35]. As CH-CA-Spe nanogels can be freely changed in size, structure, and surface modification, it is expected to have a synergistic effect when combined with immunotherapy.

## 3. Nanogel and Regenerative Medicine

Regenerative medicine can be expected to use nanogel as a carrier and to deliver drugs to lesions. To prepare macro-sized gels, CHP nanogels were modified with acryl or acryloyl groups and cross-linked with each other. This macro-sized hydrogel was called a NanoClik gel [1]. NanoClik gels were used in regenerative medicine as the material for a drug delivery system and tissue engineering. In the application of drug delivery, cytokines for bone regeneration were encapsulated in NanoClik gels and released from lesions. In tissue engineering, several kinds of cells, including stem cells, were attached to a NanoClik gel, and three-dimensional cell-based materials were built in vitro and transplanted into lesions in vivo.

### 3.1. Growth Factor Delivery

Bone morphogenetic protein-2 (BMP-2) has already been applied clinically in the United States, but there are still problems, such as the high dosage required, cost, and side effects (gingival swelling and heterotopic ossification). Using nanogels or NanoClik gels, it becomes possible to function stably with a small amount and solve these problems locally.

In previous studies, BMP-2 and prostaglandin E were encapsulated in NanoClik gels and embedded in a mouse cranial defect model. Four weeks later, bone regeneration was improved from observation using a micro-CT. By using a NanoClik gel-based scaffold containing a prostaglandin E2 receptor-specific agonist in combination with BMP-2, it was confirmed that even a low dose of BMP-2 promoted bone regeneration [36]. By co-delivering fibroblast growth factor-18 (FGF-18) and a low dose of BMP-2 using NanoClik gels, it was also revealed that well-developed bone formation was promoted.

Bone repair by the combination of BMP-2 and FGF-18 was superior to BMP-2 alone in terms of both quality and quantity due to M2 macrophage activation by FGF-18. These findings indicate that the synergistic effect of different types of growth factors will expand the possibilities of bone tissue engineering using NanoClik gels [37]. Although BMP-2 has been applied clinically in orthopedics and dentistry, it is not widely used because of side effects such as ectopic ossification, inflammation, and tumor formation [38]. All of these are caused by excessive amounts of BMP-2. The combination of FGF-18 and NanoClik gels can be expected to reduce side effects by allowing BMP-2 to act effectively at low doses.

### 3.2. Tissue Engineering

After approximately 2015, NanoClik gels were applied mainly using an acryloyl group-modified cholesterol-bearing pullulan (CHPOA) nanogel, in which a CHP nanogel was substituted for an acryloyl (OA) group and thiolated PEG [39]. This was freeze-dried to give a continuous porous structure with a diameter of several hundred micrometers and was called a “Nanogel-cross-linked porous: NanoCliP gel”. To clarify the adhesion between NanoCliP gels and cells, actin filaments (F-actin) were stained with fluorescently labeled phalloidin, and their morphology was evaluated using a confocal laser microscope (CLM). It was confirmed that the cells on the NanoCliP gel had spread widely and adhered strongly. These results suggested that the NanoCliP gel was suitable as a scaffold material for a three-dimensional culture because of its high cell adhesiveness.

Subsequently, a NanoCliP gel was freeze-dried to develop a NanoCliP-FD gel, which enabled it to be stored as a solid for a long time and absorb water-containing cells. This NanoCliP-FD gel was used to create a three-dimensional artificial structure based on osteoblasts for bone regeneration.

**Figure 2:**
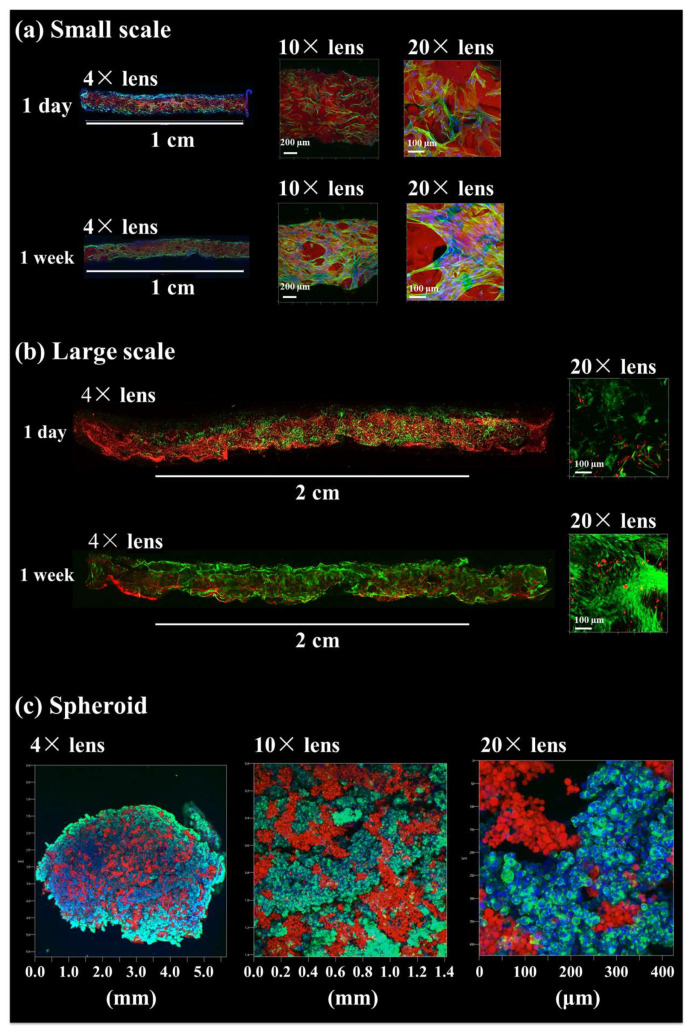
Observation of cells adhered on NanoCliP-FD gels by CLM. (**a**) Fibroblasts adhered to small-scale NanoCliP-FD gels prepared using a similar method described in a previous study [40]. Blue (nucleus), green (F-actin), red (gel). (**b**) Fibroblasts adhered to large NanoCliP-FD gels. Blue (live cell), red (gel). (**c**) HepG2 cells adhered to NanoCliP-FD microspheres on a large scale. Blue (nucleus), green (F-actin), red (gel).

Furthermore, in a previous study, osteoblasts were prepared from fibroblasts by directly reprogramming genes. The directly reprogramed osteoblasts incubated on NanoCliP-FD gels produced a calcified bone matrix and improved bone regeneration in vivo [40]. A similar experiment was carried out in a previous study, and the CLM observation results are shown in Figure 2a. In the latest study, the scale-up of the three-dimensional cell-culture system using NanoCliP-FD gels was studied. In this previous report [40], a NanoCliP-FD gel was prepared in a hematocrit capillary tube with an inner diameter of 1.1 mm. A NanoCliP gel with a thickness of ~1 mm was obtained. In the latest study, the gel was made in a new silicon rubber mold (2 mm thick and 250 mm long).

In addition, when this gel was coated with fibronectin, human-derived fibroblasts adhered. A scaled-up NanoCliP-FD gel was obtained, as shown in Figure 2b. The scaled-up gel also showed an increase in the number of cells after 1 day and 7 days of culture; this suggested that NanoCliP-FD gels can be freely changed in size and shape by preparing the appropriate template. Therefore, a three-dimensional culture of cells in the desired shape is possible. This is because the reaction between CHPOA and thiol-terminated PEG does not occur sufficiently when mixed at room temperature. Furthermore, the reaction was strongly improved under mild heating conditions of 37 °C, and an important feature was that macroscopic gel volume change did not occur before and after the cross-linking reaction.

In other previous studies, micrometer-sized NanoClik gels were prepared by emulsion-mediated cross-linking reactions [41]. The diameter of the NanoClik microspheres was ~10 µm. In the subsequent study, fibronectin was coated onto, and bone marrow-derived mesenchymal stem cells were incubated with, these NanoClik microspheres and prepared in wells of porous multi-well plates to produce hybrid spheroids of ~1.4 mm in diameter [42].

Next, there was a scale-up of the development of spheroids using NanoClik microspheres. Larger-sized spheroids were produced by culturing them using a microgravity-environment cell-culture device (Zeromo, Kitagawa Iron Works, Hiroshima, Japan). This device can control the gravity received by NanoClik microspheres. Cells are made uniform in all *xyz* directions by three-dimensional rotation. Human hepatocellular carcinoma cell lines (HepG2 cells) were cultured with NanoClik microspheres for 1 day in a microgravity-environment cell-culture device. Spheroids with a diameter of ~5.5 mm were obtained, as shown in Figure 2c. It was found that the NanoClik microspheres were arranged to fill the space between cells and, compared with spheroids consisting of only cells, they are expected to prevent hypoxia caused by cell aggregation. We obtained spheroids of a larger size than in the previously reported study.

Recently, attention has been focused on mini-organs called “organoids” [43,44,45], which can be created by embedding stem cells in an extracellular matrix (ECM) called a Corning^®^ Matrigel^®^ (a solubilized basement membrane secreted by mouse sarcoma cells [46,47]) and culturing them under appropriate conditions. Organoids are anatomically and functionally similar to organs and are in increasing demand for disease modeling and drug discovery.

However, the molecular composition of Matrigel has not been fully explained, there is large lot-to-lot variation, and there are problems such as a lack of stable supply. Additionally, Matrigel has ethical concerns because it is derived from a different species, and there are many problems with its use in regenerative medicine. If NanoClik gels derived from natural polysaccharides, such as Matrigel, can replace the functions of ECMs, safety and ethical issues can be overcome. They can be expected to be applied not only to organoids but also to regenerative medicine such as spheroids. How NanoClik gels are involved in cell migration, cell behavior, and polarity signaling in organoid cultures is unclear and needs to be clarified.

### 3.3. Bone Regeneration

Recently, a biological apatite with a 3D structure was produced using NanoCliP-FD gels. Mesenchymal stem cells (MSCs) were cultured three-dimensionally on a NanoCliP-FD gel scaffold. The resulting cultured bone tissue was found to be composed of biological apatite, which is more crystalline than a collagen scaffold [48]. The NanoCliP gel was used as a scaffold material for cell transplantation and was transplanted into the bone defect of mice together with MSCs. Infrared spectroscopic analysis using synchrotron light, which has sensitivity and high spatial resolution, was used for the analysis. Micro-CT analysis confirmed significant bone regeneration in the NanoCliP gel-implanted group compared with the untreated group [49]. 

**Figure 3:**
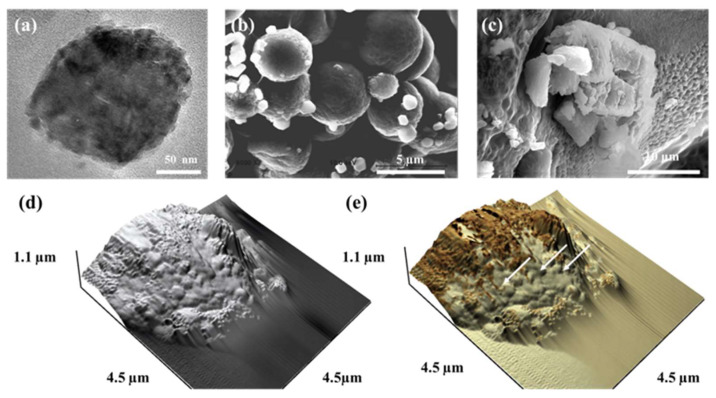
Observation of biological apatite on a NanoCliP gel using electron microscopy. Transmission electron microscopy (TEM) and scanning electron microscopy (SEM) images are shown. Matrix vesicles (**a**), with a grain size of ~200 nm, are the starting points of calcification, calcified globules formed with the progress of calcification, and apatite crystals are observed inside the NanoCliP (**b**,**c**). Apatite crystals and collagen (arrow) are observed (**d**,**e**).

Using Raman spectroscopy, a non-destructive and non-invasive method for analyzing the molecular structure of materials, we reconstructed the Raman bands of molecules belonging to the constituents of bone (collagen, PO_4_^3−^, and CO_3_^2−^) and obtained Raman imaging. As a result, in the regenerated bone tissue implanted with a NanoCliP gel, a sharp peak for ν1PO_4_^3−^ (965 cm^−1^), attributed to hydroxyapatite (HAP), was confirmed, and mature bone tissue was formed. Electron microscopy (Figure 3a–c) and scanning probe microscopy of the surface and interior of the NanoCliP gel revealed not only matrix vesicles, which are the origin of calcification, but also various vesicles. We confirmed the presence of various sizes and types of calcium phosphate compounds (Figure 3d,e).

Due to the chemical properties of the surface of NanoCliP gel, the accumulation of calcium ions promotes the growth of HAP crystals along the *c*-axis, which exerts a strong mechanical function. This is thought to form a bone tissue that has both “rigidity” and “elasticity.” Carbonic apatite is absorbed by osteoclasts and is known to have high bone-replacement and bone-forming ability. It is thought that good-quality bone tissue can be obtained by remodeling.

A NanoCliP gel induces the formation of regenerated bone tissue with both high stiffness and elasticity in both in vitro and in vivo systems. It is hoped that it will bring benefits. To date, MSCs have been used as a source of osteoblasts, but they cause problems such as high invasiveness at the time of collection and a limited number of obtained stem cells in some cases. In addition, induced pluripotent stem cells (iPS cells) risk tumorigenesis due to residual undifferentiated iPS cells. Conversely, it has been reported that some types of functional somatic cells, such as cardiomyocytes [50,51], neurons [52,53], hepatocytes [54], skeletal muscle stem cells [55], and brown adipocytes [56], have been directly induced from differentiated fibroblasts without passing through the pluripotent stage. 

These methods have been recently known as direct conversion or direct reprogramming. Direct conversion has some advantages over using iPS cells in terms of convenience, speed, low cost, and low tumorigenic risk. We found that human functional osteoblasts were induced from fibroblasts by transducing defined factors [57]. However, genetic manipulations raised safety concerns and were thus not desirable for most clinical applications. So, we developed alternative methods and induced osteoblastic cells from human fibroblasts directly using only chemical compounds [58]. Direct conversion using chemical compounds is cost-effective, easy to manipulate, and much safer because it does not involve the integration of chromosomes.

**Figure 4:**
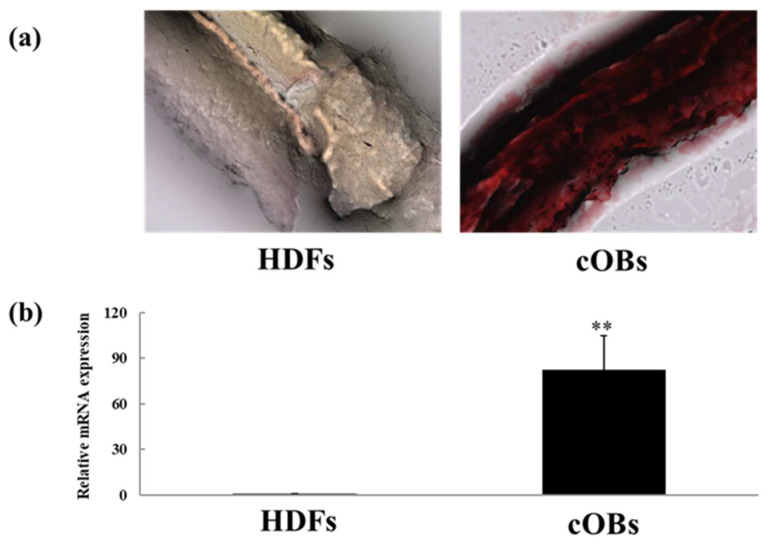
Characters of the complex of each cell and NanoCliP-FD gel. Alizarin Red S staining of the complex of each cell and NanoCliP-FD gel (magnification 40×) (**a**). ALP mRNA expression of the complex of each cell and NanoCliP-FD gel (**b**). ** *p* < 0.01 vs. HDFs. HDFs: human dermal fibroblasts, cOBs: chemical compound-mediated directly converted osteoblasts.

As the next step, we tried the three-dimensional direct conversion of human osteoblasts using a NanoCliP-FD gel [58]. This route was chosen because a NanoCliP-FD gel can be molded into various shapes, and by combining it with direct conversion, we could provide safe, secure, and high-quality tailor-made bone regenerative medicine. It was confirmed that the cells could attach to the surface of NanoCliP-FD gel, proliferate well, and produce large amounts of calcified bone matrix (Figure 4). The calcified bone matrix produced on a NanoCliP-FD gel also contained carbonate apatite, which is easily absorbed by osteoclasts and has high bone-replacement properties, so bone remodeling reactions could be expected [49,58].

NanoCliP gels can also be molded into various shapes, and by combining them with direct conversion, we could provide safe, secure, and high-quality tailor-made bone regenerative medicine that does not contain exogenous genes or components derived from different animals (xeno-free) [58].

### 3.4. Cartilage Regeneration

Articular cartilage is essential for improving joint gliding and absorbing shock when weight is applied. Articular cartilage is damaged when a large load is applied through sports or external forces. Articular cartilage has no nerves or blood vessels that supply nutrients and lacks repair cells, making it difficult to heal naturally [59]. Therefore, there is no radical treatment for osteoarthritis (OA), a degenerative disease of cartilage.

In recent years, with the development of tissue engineering, there has been active development of regenerative treatments that transplant cartilage tissue or chondrocyte sheets cultured in vitro. Autologous cartilage is sometimes used as a source of cartilage [60], but invasiveness during tissue collection poses a problem. Multifamily cartilage therapy, in which cartilage tissue that is discarded during surgery for polydactyly patients is transplanted, has also been reported [61,62]. Abe et al. reported that chondrocytes and cartilage tissue were differentiated from iPS cells and allografted and that iPS cell-derived cartilage was engrafted [63]. Conversely, as mentioned above, although iPS cells have multipotency, they carry the risk of tumorigenesis [64,65,66]. Therefore, culture technologies that reduce the risk of tumor formation have been developed, including the direct reprogramming method that creates cartilage without using iPS cells [67,68] and the “three-step method” that differentiates iPS cells into cartilage via MSCs [69].

Human periodontal ligament stem cells (hPLSCs), which are MSCs with the ability to differentiate into various cells (bone, cartilage, and nerve) [70,71], have relatively many opportunities to be collected and can be collected relatively easily. When MSCs are differentiated into cartilage tissue, dedifferentiation occurs in planar culture, so a three-dimensional culture is sometimes performed using scaffolding materials to maintain the cartilage state.

For cartilage transplantation to be successful, it is necessary to construct cartilage with excellent mechanical properties that contain abundant cartilage matrix and to evaluate the quality of the cartilage matrix at the molecular level. However, scaffold materials suitable for cartilage culture are poorly understood. We evaluated NanoCliP gels using a spectroscopic analysis of the molecular structure of constructed cartilage tissue and evaluated whether NanoCliP could become a basic technology for new cartilage regeneration therapy [72]. hPLSCs were seeded onto NanoCliP gels and cultured three-dimensionally under cartilage differentiation induction.

After 14 days, the NanoCliP cartilage tissue and culture supernatant were subjected to immunological analysis (MIA: melanoma inhibitory activity, a chondrocyte differentiation marker [73]) and spectroscopic analysis (Raman spectroscopy and synchrotron radiation [SR] FT-IR) to evaluate the expression of the cartilage matrix (glycosaminoglycan and collagen). Overall, 60–80% of cartilage is water, and the remaining 20% is the cartilage matrix (the substance between the cells). The cartilage matrix is composed of collagen and proteoglycans, which are sugars bound to proteins; proteoglycans include glycosaminoglycans (hyaluronic acid, chondroitin sulfate, and heparan sulfate). Raman spectroscopy and FT-IR spectroscopy can be used for analysis [74,75,76,77,78,79,80,81].

By culturing MSCs on a NanoCliP, we found that differentiation into chondrocytes is promoted more than atelocollagen, and cartilage tissue rich in a cartilage matrix such as hyaluronic acid is constructed. In addition, the spectroscopic analysis revealed that the protein secondary structure formed on NanoCliP gels had a relatively higher proportion of α-helical conformations than random coil conformations and had robust and stable collagen. In particular, it is known that cartilage tissue in osteoarthritis has a relatively large amount of random coil conformations. In contrast, healthy cartilage has a relatively large amount of α-helical conformations [82]. Therefore, NanoCliP gels, which can construct α-helix-rich cartilage tissue, are an ideal scaffold material.

By serving as a source of glucose, NanoCliP gels are thought to increase hyaluronic acid synthesis in chondrocytes via hyaluronan synthase. In particular, as cartilage does not have blood vessels, it is logical that the NanoCliP gel can supply glucose. By combining NanoCliP gels and spectroscopic analysis, it was possible to provide high-quality cartilage regeneration treatment.

If the cultured cartilage tissue could be molded with a 3D printer and transplanted to the bone/cartilage defect [83], this may be a new treatment method for osteochondral diseases.

### 3.5. Biodegradability, Antibacterial Activity, and Mechanical Strength in Hydrogels and Polymers

Hydrogel design considers performance in terms of structural integrity, biocompatibility, biodegradability, antibacterial activity, and mechanical strength. Polylactic acid (PLA) and polyglycolic acid (PGA) are common bioabsorbable polymers that are widely used clinically. In vivo, absorbable sutures made of PGA lose half their material strength in 2 weeks and nearly 100% in 4 weeks. It is estimated that it takes 4–6 months for the thread to be completely incorporated into the body [84]. Moreover, the collagen/HAP scaffold material did not degrade and maintained its morphology for more than 6 months in vivo [85].

Sato and Adachi et al. implanted the NanoCliP-FD gel into bone defects in mice and evaluated bone regeneration [40,49]. These experiments had short-term experimental schedules (approximately 4 weeks), and the residual gel was observed in the femoral bone defect. The NanoCliP-FD gel maintained its gel form for more than 4 weeks, even in the femur, which is susceptible to mechanical stress, and was found to have sufficient strength. These results indicated that the NanoCliP-FD gel is expected to be used in bone and cartilage regenerative medicine. It requires flexibility and fracture resistance that can follow the movements of organs and living tissues. As a future experimental topic, it will be necessary to observe the progress over 1 year or more to evaluate the biodegradability of the NanoCliP-FD gel.

When biodegradable polymers are implanted into a living body for a long time, it is necessary to be careful about infection. Conjugating the non-biodegradable polymer poly(methyl methacrylate) (PMMA) with antimicrobial agents has been established as a treatment for periprosthetic joint infection [86]. Several antibacterial agents have been incorporated into dental adhesives and cement to render them anticariogenic [87]. Conversely, research on imparting antibacterial properties to the biodegradable polymer PLGA/β-TCP composite is still in the development stage [88]. The biofilm formation inhibition and antibacterial properties of NanoCliP-FD are not well known. Regarding the practical application of NanoCliP-FD, one option is to use it with materials or drugs that impart antibacterial properties without impairing its original physiological activity and physical properties.

### 3.6. Ingredients

Collagen and gelatin, a common scaffolding material, are made from bovine dermis. In recent years, there has been a movement to refrain from eating meat from an environmental protection and sustainability perspective. Further, social interest in alternative meats, such as soybean meat and cultured meat, and in veganism has increased. Food production using livestock is said to have a large environmental impact because livestock emit greenhouse gases and use water resources and land intensively. Therefore, switching from livestock-based resources has become important in realizing a sustainable society.

Microorganisms that produce naturally derived bio-based polymers [89] are attracting attention, as these are a sustainable resource and do not require large areas of land compared with cows. *Streptococcus salivarius*, a type of cavity-causing bacterium, can create unbranched crystalline α-1,3-glucan esters with high thermal stability and a high melting temperature from the renewable raw material sucrose. It can be used as a source of glucose monomers, resulting in an environmentally friendly one-pot water-based reaction [89]. The raw material for nanogels is pullulan, a water-soluble polysaccharide produced from starch syrup by *Aureobasidium pullulans*, a type of filamentous fungus [90]. It is tasteless, odorless, easily soluble in water, highly stable, and has lubricating properties. Medical technology based on nanogels derived from natural polysaccharides is expected to contribute to creating a sustainable society.

## 4. Conclusions

A CHP nanogel has been used since 2004 in clinical trials as a DDS carrier, and its effectiveness has been demonstrated [12]. In particular, the therapeutic effect on esophageal cancer has been remarkable and clinical trials are underway. Progress is being made in developing a COVID-19 nasal vaccine in which a CHP nanogel and antigen are administrated nasally [3,13].

Furthermore, progress is being made in developing a highly safe COVID-19 vaccine made from a hyaluronic acid-derived nanogel that does not require a cold-chain and does not contain PEG. Using Nanogel as a carrier, it will be easily possible to vaccinate patients and children who have PEG allergies. The vaccine is expected to be used widely even in developing countries with a poor medical infrastructure.

Bottom-up gel fabrication using nanogels as building blocks as a technology, “nanogel engineering”, is expected to be applied to DDSs and scaffolding materials for regenerative medicine, etc. CHP nanogel scaffolds have the ability to mimic natural tissue structure and function and may be one of the most effective and viable therapeutic options [39,40,41,42,48,49,58,72]. Conversely, there are many uncertainties in the clinical application of CHP nanogel scaffold materials. Considerable further research is required to ultimately reach human trials and clinical trials. It is thought that this will lead to the development of excellent biomaterials that solve various medical problems, including medical care for the elderly.

## 5. Patents

The following applied patent provides details about the scaffold materials: PCT/JP2019/033678.
